# A Study on the Prediction of Compressive Strength of Self-Compacting Recycled Aggregate Concrete Utilizing Novel Computational Approaches

**DOI:** 10.3390/ma15155232

**Published:** 2022-07-28

**Authors:** Jesús de-Prado-Gil, Covadonga Palencia, P. Jagadesh, Rebeca Martínez-García

**Affiliations:** 1 Department of Applied Physics, Campus de Vegazana s/n, University of León, 24071 León, Spain; c.palencia@unileon.es; 2 Department of Civil Engineering, Coimbatore Institute of Technology, Coimbatore 638056, Tamil Nadu, India; jaga.86@gmail.com; 3 Department of Mining Technology, Topography, and Structures, Campus de Vegazana s/n, University of León, 24071 León, Spain; rmartg@unileon.es

**Keywords:** artificial neural network, self-compacting concrete, recycled aggregates, compressive strength, Levenberg–Marquardt, Bayesian regularization, Scaled Conjugate Gradient Backpropagation

## Abstract

A considerable amount of discarded building materials are produced each year worldwide, resulting in ecosystem degradation. Self-compacting concrete (SCC) has 60–70% coarse and fine particles in its composition, so replacing this material with another waste material, such as recycled aggregate (RA), reduces the cost of SCC. This study compares novel Artificial Neural Network algorithm techniques—Levenberg–Marquardt (LM), Bayesian regularization (BR), and Scaled Conjugate Gradient Backpropagation (SCGB)—to estimate the 28-day compressive strength (f’c) of SCC with RA. A total of 515 samples were collected from various published papers, randomly splitting into training, validation, and testing with percentages of 70, 10 and 20. Two statistical indicators, correlation coefficient (R) and mean squared error (MSE), were used to assess the models; the greater the R and lower the MSE, the more accurate the algorithm. The findings demonstrate the higher accuracy of the three models. The best result is achieved by BR (R = 0.91 and MSE = 43.755), while the accuracy of LM is nearly the same (R = 0.90 and MSE = 48.14). LM processes the network in a much shorter time than BR. As a result, LM and BR are the best models in forecasting the 28 days f’c of SCC having RA. The sensitivity analysis showed that cement (28.39%) and water (23.47%) are the most critical variables for predicting the 28-day compressive strength of SCC with RA, while coarse aggregate contributes the least (9.23%).

## 1. Introduction

The most significant component of the building business is concrete. Because durability has become one of the most critical issues in building reinforced concrete structures with long service lives and with the development of construction technologies in recent years, it is necessary to manufacture well-designed concrete as a robust material for construction [1,2,3].

Concrete is the most used building material globally. Because numerous types of concrete with different admixtures are being created, the understanding of advanced concrete design procedures has expanded [4]. One of the outcomes of advanced concrete is self-compacting concrete, which was developed in Japan (1980) to produce high-strength and durable concrete structures [5,6].

The fundamental difference between self-compacting and ordinary concrete is the quantities of components used in the mixing process. SCC is recognized as the era’s most creative concrete, with the ability to self-settle in building zones without vibratory power. Self-compacting concrete sinks under its weight by following a flowing course [7]. SCC is noted for being novel since it may be utilized in dense regions where concrete is difficult to come by [8].

The unprecedented growth of the building sector through the decades has resulted in a disproportionate use of natural properties; demolition, and construction waste (DCW) has gathered in a large volume, and landfilling recycled aggregates (RA) can cause environmental destruction [9,10,11]. The construction sector in the European Union has experienced an exponential expansion in recent decades, and because of this growth, DCW output has increased. With the building sector’s rapid growth, the demolition rates are growing daily, necessitating the effective reuse of DCW [12,13,14]. Fine aggregates (sand) and coarse aggregates (stone) make up most of the concrete, accounting for roughly 75% of the overall volume [15,16].

Simultaneously, natural resources are decreasing because of rapid modern urbanization. Mountains, the principal supply of high-quality aggregates, rapidly diminish [17,18]. As a result, numerous nations worldwide have experienced natural disasters [19]. On the other side, many structures are demolished every year to earthquakes or because they have outlived their usefulness [20,21]. So, yearly, a significant amount of building trash is produced worldwide.

Recent advances in concrete material research have paved the road for alternative materials as long-term replacements for traditional ones [22,23,24,25]. Using recycled aggregates in self-compacting concrete is the first and most sustainable revolution to counteract such problems. Recycled aggregates (RA) are made by reprocessing mineral waste materials, with building and demolishing debris being the most common source. For the same amount of aggregate, recycled aggregate concrete (RC) underperforms natural aggregate concrete (NC). Various admixtures can compensate for mechanical and durability deficiencies [26,27,28]. As a result, a good mix design is essential for concrete manufactured with RA to have the needed properties [29,30,31,32].

The second technique is to skip all the experimental work, decreasing environmental deterioration and natural resource waste. Many researchers are focusing on soft computing strategies these days. These computational approaches such as machine learning or artificial neural network techniques have lately emerged as practical tools for modeling and estimating various issues, notably in concrete modeling characteristics [33]. The human brain, which comprises billions of neurons, is a model for artificial neural networks. Similar to how a human learns through experience, an artificial neural network (ANN) uses data to anticipate variables [34].

In ANN, different algorithms are there. In this study, three algorithms were selected based on their performances. The Levenberg–Marquardt algorithm often requires more memory, but it is faster. Even though Bayesian regularization requires more time, it can produce strong generalization for challenging, small, or complex datasets. The Scaled Conjugate Gradient Backpropagation algorithm, on the other hand, utilizes less memory than the preceding ones.

The primary purpose of this study is to use artificial neural networks to validate and forecast the compression strength of CC utilizing RA. Different techniques, such as Levenberg–Marquardt (LM), Bayesian regularization (BR), and Scaled Conjugate Gradient Backpropagation (SCGB), are developed and compared for this purpose.

## 2. Literature Review

### 2.1. Artificial Neural Network

In deep learning, artificial neural networks (ANN) are a crucial method. A subset of machine learning called deep learning (DL) enables the computation of multilayer neural networks. In contrast to artificial intelligence’s primary objective of enabling machines to mimic human behavior, machine learning employs statistical techniques to enable computers to grow over time. The key contrast between machine learning and deep learning is that in DL, pattern extraction and categorization are completed automatically. However, with machine learning, feature extraction is still necessary, and the computer handles categorization and prediction [35].

An artificial neural network (ANN) is a complex mathematical and computational model that is based on the extensive biological neural network in the human brain [36]. It may increase its performance by learning from its mistakes, which is how an artificial neural network knows. It comprises a network of functions and weights that act as artificial neurons. They are usually used in artificial intelligence applications that require them to analyze difficult and complicated problems [34].

Each unique and specific algorithm can be used to run an ANN. The following sections cover LM, BR, and SCGB from a research standpoint.

#### 2.1.1. Levenberg–Marquardt Algorithm

The Levenberg–Marquardt (LM) approach iteratively seeks out the function’s least value. A multidimensional function may be expressed as the sum of squares of nonlinear real-valued functions [37,38]. This method has been applied by researchers to challenging nonlinear least-squares problems in several different domains [39]. In this algorithm, two approaches, steepest descent and the Gauss–Newton method, are merged to speed up iterations and reduce error. The algorithm transitions to the Gauss–Newton approach more rapidly than the others when the most recent result is accurate. When the outcome is incorrect, it acts as a steepest decline: slow but always capable of approximation [40]. The main advantage is that although this approach requires more memory, it takes little time.

#### 2.1.2. Bayesian Regularization

Bayesian regularized artificial neural networks (BRANNs), which can shorten or eliminate the requirement for time-consuming cross-validation, are more reliable than traditional back-propagation neural networks (BPNNs), which are less reliable [41]. Bayesian regularization transforms in the same manner as a nonlinear regression is converted into an accurate statistical problem using ridge regression. They take longer, but the model offers many advantages over challenging data [42]. The benefit of using BRANNs is that there is no requirement for a validation phase because the models are resilient [43,44]. The challenges of quantitative structure–activity relationship (QSAR) modeling include prediction, reliability, choosing the right validation sets, and optimizing network design. Empirical processes stop Bayesian criteria from being used for training, making it nearly impossible to over train [45].

#### 2.1.3. Scaled Conjugate Gradient Backpropagation

The basic backpropagation method is used to modify the weights in the direction of steepest fall or highest negative gradient. This is the method through which the performance function can degrade the quickest. Although the function descends the negative gradient the quickest, it is demonstrated that this does not necessarily indicate the quickest integration [46].

The conjugate gradient (CG) algorithms seek a direction that achieves faster convergence than the steepest descent way while maintaining the error reduction attained in the earlier stages, and this activity is known as the conjugate direction. In most CG algorithms, the step size changes with each iteration. A search is undertaken along the conjugate gradient direction to determine the step size that will minimize the performance function along that line [47].

A method other than the line search approach can also estimate the step size. The goal is to combine the model trust region approach of the LM algorithm with the CG methodology. This strategy is known as SCGB, which was initially reported in the literature by Møller (1993) [48].

Design parameters are adjusted separately for each iteration user, which is essential for the algorithm’s success. This is a substantial advantage over line-based algorithm searches [48].

## 3. Research Significance

The compressive strength of self-compacting concrete containing recycled aggregates is validated and predicted in this study using artificial neural networks. Based on the author’s best understanding of the research currently available, there has not been a significant study on using various deep learning techniques to forecast the compressive strength of SCC with RA, which marks its novelty. Several techniques, including the Levenberg–Marquardt (LM), Bayesian regularization (BR), and Scaled Conjugate Gradient Backpropagation (SCGB) algorithms, are applied for this objective. Two statistical indicators, correlation coefficient (R-Value) and mean square error (MSE), are employed for the selection of the best model among them all. Sensitivity analysis is then conducted to determine the impact of each input variable on the output variable. The present study will provide comprehensive knowledge to the readers about these three algorithms for the prediction and validation of SCC with RA.

## 4. Materials and Methods

### 4.1. Experimental Plan

Information is acquired from numerous study articles. Table 1 presents the database containing 515 samples of SCC compressive strength f’c with RA, including six variables named X1 to X8 and one output Y, i.e., compressive strength. The variables of input include Portland cement (X1), supplementary cementitious materials (X2), water (X3), FA (X4), CCA. (X5), and admixtures (X6). The database includes Sr No., which displays the overall collection of articles, the authors’ citations, the amount of information (# data) supplied by each paper, and the percentage of the total data (% data).

**Figure 1:**
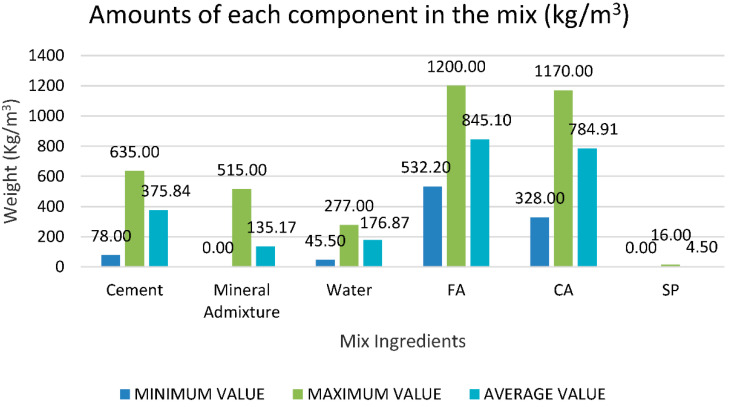
Minimum, mean and maximum values of input variables.

**Figure 2:**
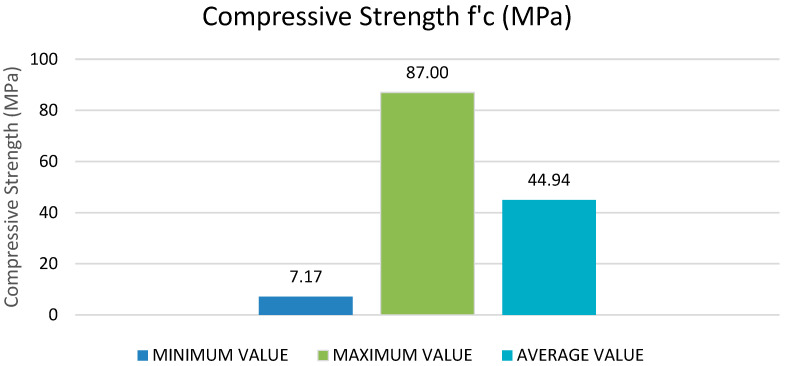
Minimum, mean and maximum values of output variables.

Table 2 illustrates the lowest, highest, and mean of specific variables as inputs (cement, supplementary cementitious materials, fine aggregate, water, coarse aggregates, and superplasticizers) and one potential output (compressive strength of recycled aggregate self-compacting concrete) based on these published research publications. Figure 1 and Figure 2 illustrate their graphical representation.

### 4.2. Data Visualization

#### 4.2.1. By Frequency Distribution

**Figure 3:**
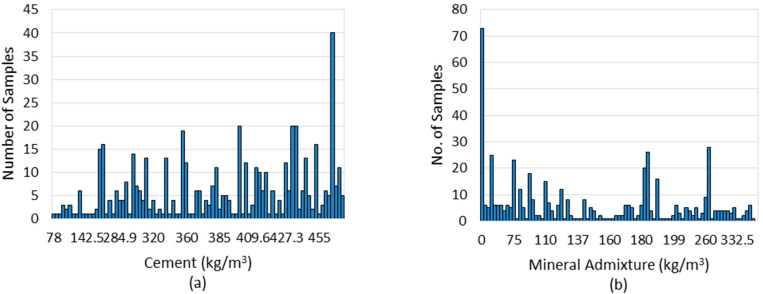

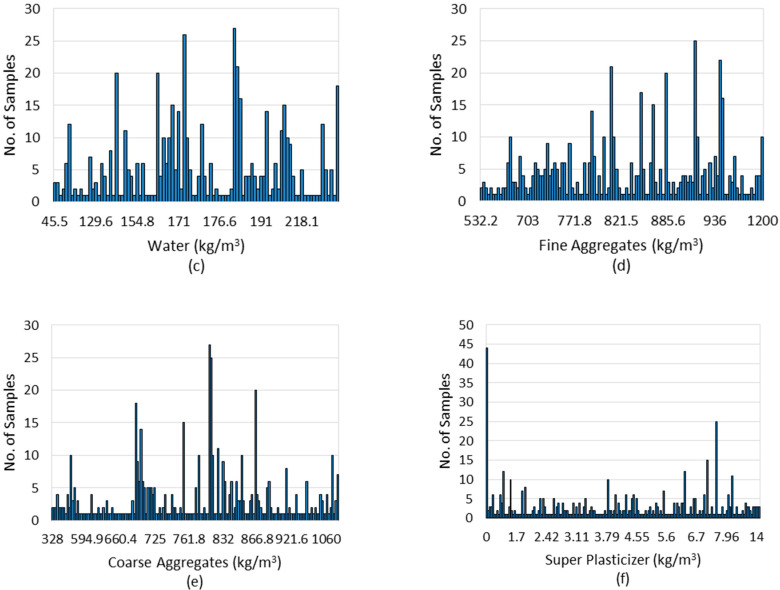
Frequency analysis (histogram) of input parameters: (**a**) Cement; (**b**) Mineral Admixture; (**c**) Water; (**d**) Fine Aggregates; (**e**) Coarse Aggregates; (**f**) Superplasticizer.

The input variables from X1 to X5 have a vast range of values, but the variable X6 has a limited range of values. The cement content (X1) ranges from 78 to 635 (kg/m^3^), with most of it being between 180 and 600 (kg/m^3^). The maximum sample number is around 40, corresponding to a cement concentration of 635 kg/m^3^. Likewise, the mineral admixture (X2) varies between 0 and 515 kg/m^3^. The water content (X3) varies from around 45 to 277 (kg/m^3^), as indicated in Figure 3. Fine aggregate or sand content (X4) ranges from 532 to 1200 (kg/m^3^), with most values between 770 and 1000 (kg/m^3^). The coarse aggregates (X5) range from 328 to 1170 (kg/m^3^), with typical values falling between 680 and 920 (kg/m^3^). The superplasticizer content (X6) is between 0 and 16 kg/m^3^. It can be seen from the figures that out of all 515 samples, everyone contributes to the respective input variable.

#### 4.2.2. By Multi-Correlation Graph (Heat Map)

**Figure 4:**
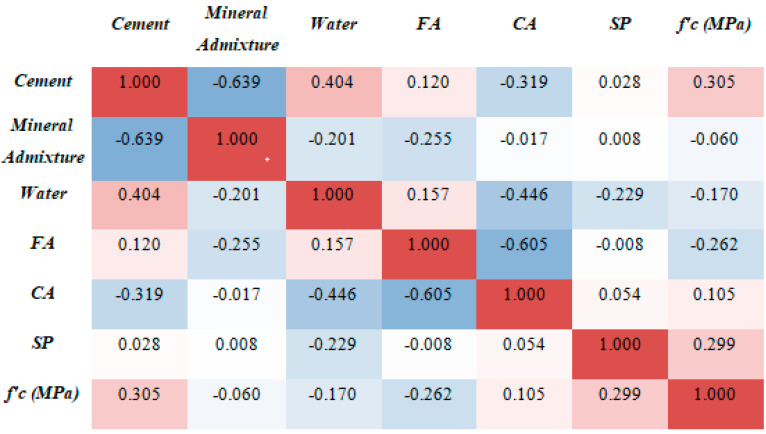
Multi-correlation graph between input and output variables.

This statistical analysis helps in the development of the predictive model by improving the accuracy of the outcome prediction. The relationship between the variables of input (fine aggregates, water, cement, admixtures, superplasticizers, and coarse aggregates) and the variable of output (compression strength) was investigated to see if there was a link [100]. The Pearson correlation matrix (heat map) is created to analyze the correlation between the independent input variables, as illustrated in Figure 4. The model’s predictions might be skewed if input variables have correlations (|R| > 0.8) that suggest multicollinearity between variables. Although several characteristics are significantly correlated, for example, cement and mineral admixtures have a correlation of −0.639, whereas CA and FA have a correlation of −0.605. However, none of the features showed a correlation (|R|) greater than 0.80, demonstrating the lack of multicollinearity [101,102].

### 4.3. Methodology of Artificial Neural Network Model

An artificial neural network is a data prediction framework based on present characteristics developed from the human mind structure known as an artificial neural network (ANN). This system is made up of neurons, which are functional blocks. Weights link neurons, which are generally chosen at random initially. Various epochs enhance or reduce the importance of a learning process to finally produce the ideal network that can predict it with fair accuracy [103].

**Figure 5:**
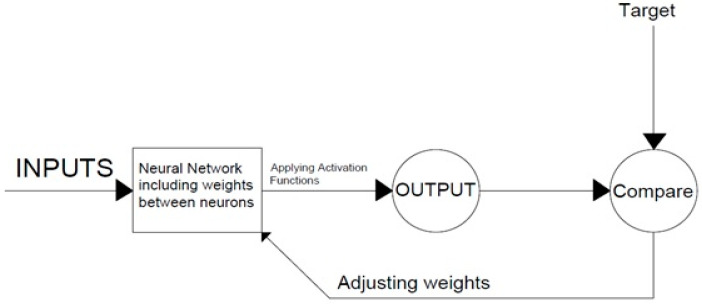
General Structure of the ANN.

As an outcome, a trained neural network may produce the intended output by receiving the inputs and considered the updated weights, as shown in Figure 5. The system becomes stronger by computing the error and comparing the required input and output. ANN includes three steps: training, validation, and testing. The model is run repeatedly throughout the training phase until the desired outcome is obtained. Errors from the validation stage are discovered during training [104]. The fact that the machine learning model improves with time implies that the prediction model’s accuracy may be increased and that the projected outcomes are dependable. Nonlinear activation functions such as sigmoid (tansig and logsig) are commonly utilized due to their exceptional responsiveness [105].

When developing an ANN model, several aspects must be considered. The initial step is to choose the most optimal ANN model structure. The data must then be entered into the chosen ANN model regarding input and output. After that, the experience must be used to select the activation function, number of layers, number of hidden layers, and number of neurons in each layer [106,107].

**Figure 6:**
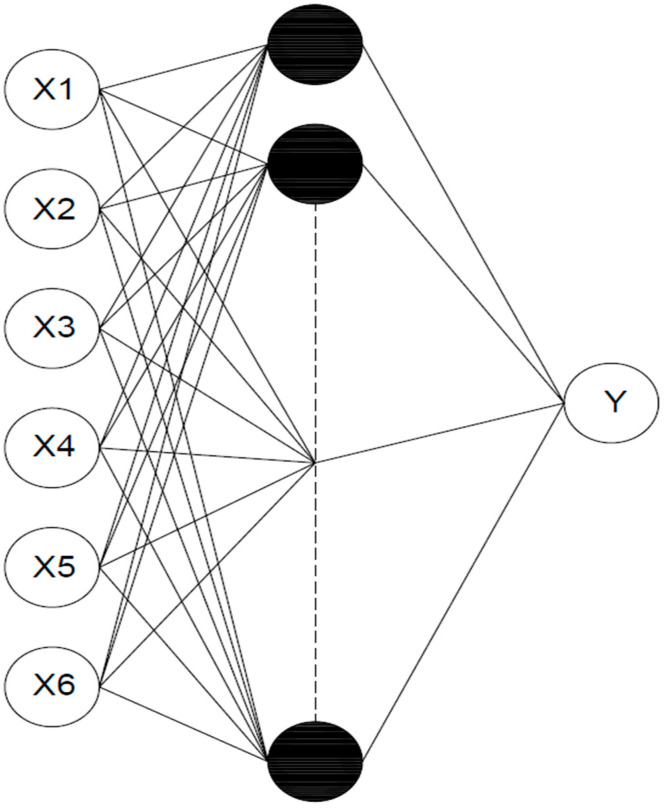
Architecture of the ANN model.

Considering Table 1 and Table 2, the network in this study comprises six inputs, one output variable, and a single hidden layer. Cement, admixtures, water, fine and coarse aggregates, and superplasticizer are all variables in the input layer. The output variable was chosen as the compressive strength of the self-compacting concrete with recycled aggregates. This research employs a feed-forward backpropagation neural network. Figure 6 shows the architecture of the present ANN research.

The Levenberg–Marquardt (LM), Bayesian regularization (BR) and Scaled Conjugate Gradient Backpropagation (SCGB) techniques were employed and compared in this work. MATLAB software was used to design and run the network.

Even though the Levenberg–Marquardt method is quicker, it frequently uses more memory. As seen by an increase in the mean square error of the validation samples, training automatically ends when generalization stops improving. However, even if Bayesian regularization takes more time, it can yield good generalization for complicated, minor, or challenging datasets. Training comes to an end because of adaptive weight loss (regularization). On the other side, the Scaled Conjugate Gradient Backpropagation technique uses less memory than the previous ones. When generalization stops improving, training automatically terminates, as seen by an increase in the mean square error of the validation samples [48,108].

**Figure 7:**
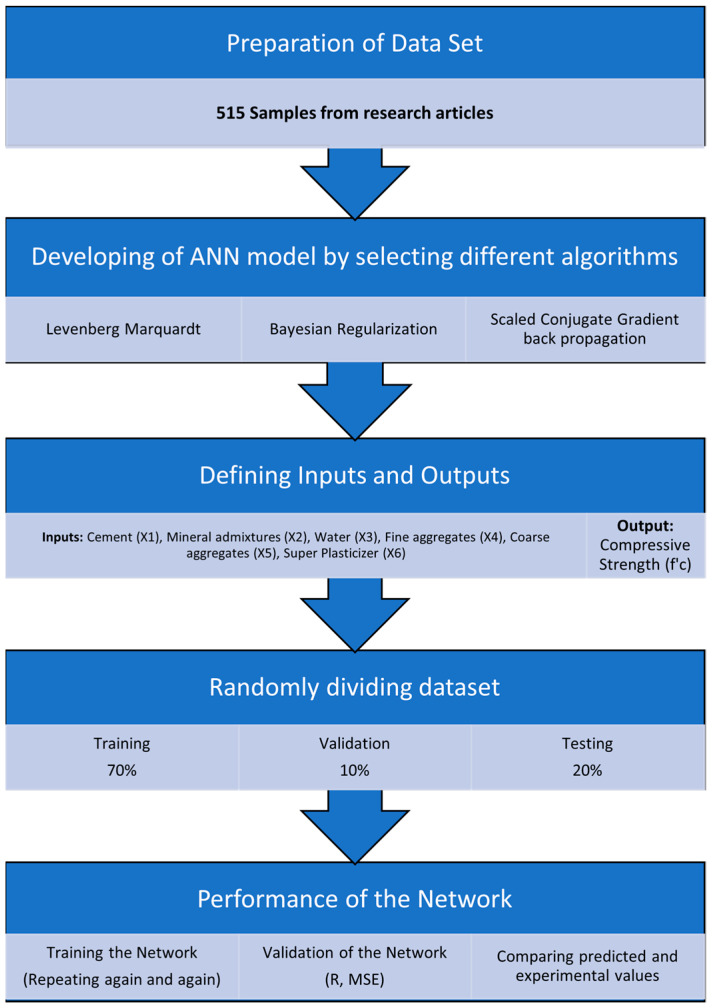
Methodology of research work.

The three phases of the network are training, validation, and testing. Table 3 shows the data splitting for the model’s training, validation, and testing. Seventy percent of the data are chosen for training, while the remaining ten percent and twenty percent are selected for the validation and testing stages. In the 1st phase, ten neurons were chosen for the hidden layer. Based on its percentage, the network randomly selected 360 samples for training, 52 samples for validation, and 103 samples for testing. For Bayesian regularization (BR), validation is not required; hence, the training and testing samples are 412 and 103, respectively. This is so because regularization typically involves validation; however, BR techniques already include an in-built type of validation. The study work’s methodology is displayed in Figure 7.

### 4.4. ANN Network Model Assessment

Mean squared error (MSE) and coefficient of correlation (R-Value) were used to assess the models’ performance [109,110], as shown in Equations (1) and (2), respectively.

(1) $$R=\frac{\sum_{i=1}^{n}(\mathrm{exi}-\bar{\mathrm{exi}})(\mathrm{moi}-\bar{\mathrm{moi}})}{\sqrt{\sum_{i=1}^{n}{(\mathrm{exi}-\bar{\mathrm{exi}})}^{2}\sum_{i=1}^{n}{(\mathrm{moi}-\bar{\mathrm{moi}})}^{2}}}$$

where exi, moi, $\bar{\mathrm{exi}}$, and $\bar{\mathrm{moi}}$ are the experimental values setup and model domain.

(2) $$\mathrm{MSE}=\frac{1}{n}\sum {\left(y_{i}-{\hat{y}}_{i}\right)}^{2}\;$$

where n = number of data points, $y_{i}$ = observed values and ${\hat{y}}_{i}$ = predicted values.

Regression is acknowledged to be the most important metric for determining the accuracy of a network’s overall accuracy. R-values are used to assess the relationship between outputs and projected targets. The R-value of a strong association is 1, whereas the R-value of a random relationship is 0 [109].

Mean squared error is the average squared disparity between outputs and objectives. It is preferable if the value is as low as possible. If the value is 0, there is no error.

## 5. Results and Discussion

The model was performed using three different algorithms: LM, BR, and SCG with the results compared and explained below.

### 5.1. Levenberg–Marquardt Algorithm

**Figure 8:**
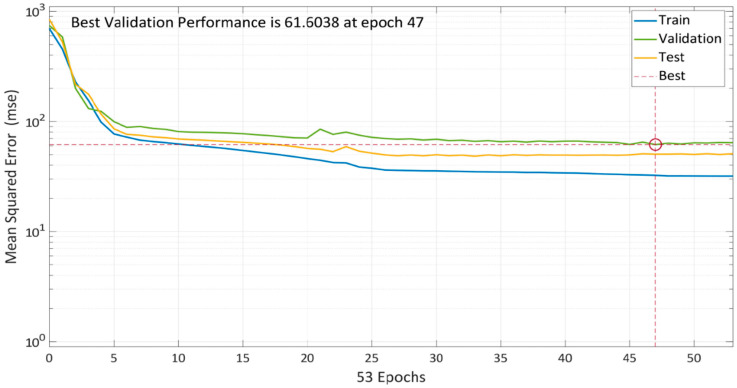
Model performance using LM algorithm.

To find the best model, the algorithm is continuously trained. The model’s performance with 10 total neurons is shown in Figure 8. Multiple colored lines make up the plot, which stand for training, validation, and testing. To prevent data overfitting, the model starts with a high MSE and subsequently decreases it based on validation criteria. After 47 epochs, the training error started to decrease, but validation and testing errors were increasing. As a result, the model training process concluded after five further epochs, and at the 47th iteration, an optimized model with the lowest MSE of 61.6038 was created.

**Figure 9:**
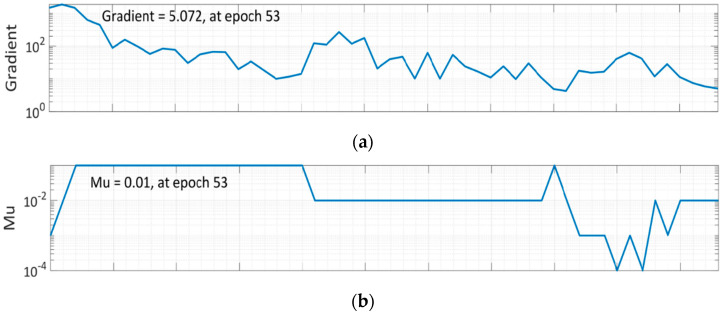

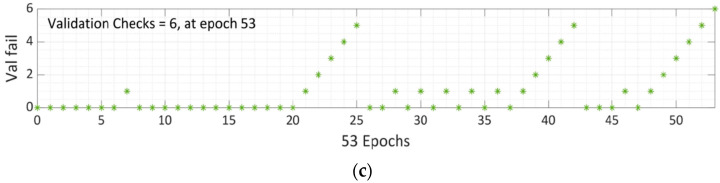
Training State of LM Algorithm: (**a**) Gradient graph at epoch 53; (**b**) Mu graph at epoch 53; (**c**) Validation checks graph at epoch 53.

Epoch 47 is found out to be most suitable option for LM algorithm network training because, while the mistakes in training data decline over time, the errors in validation and test data rise. In the Levenberg–Marquardt method, Mu is the learning rate, and 0.01 was chosen after specific iterations (Figure 9b). The training process has been halted after six validation failures.

**Figure 10:**
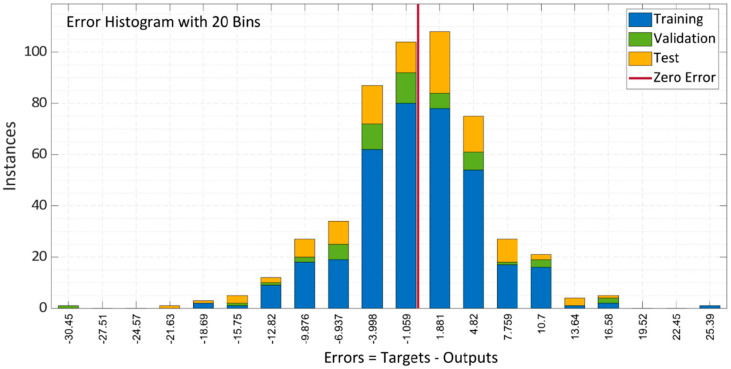
Error histogram of LM algorithm model.

Figure 10 depicts the model error histogram for training, validation, and testing. The bars converge to the centered red line, which indicates zero error in the graph. These results concluded that the LM model is appropriate in forecasting the results of the compressive strength of SCC with RA.

**Figure 11:**
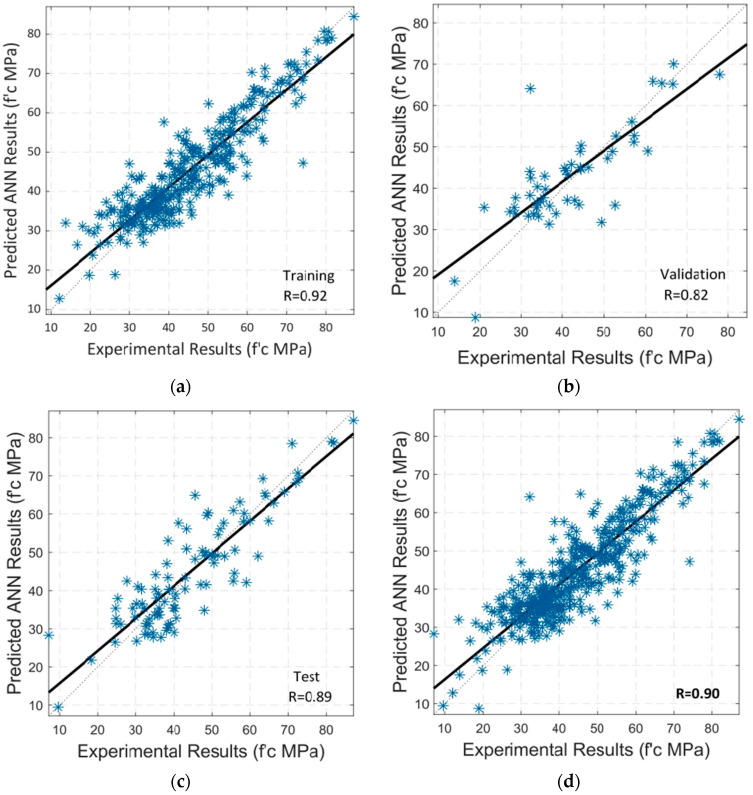
Regression analysis by using LM algorithm between experimental and predicted compressive strength: (**a**) Training; (**b**) Validation; (**c**) Testing; (**d**) Overall Dataset.

Regression analysis is then performed. Figure 11a–c illustrate the correlation of training, validation, and testing between the parameters of input and output of the model. Figure 11d depicts the model’s overall correlation. A black-colored linear line is presented in each case. The total R-value of 0.86 indicates that the correlation is quite close to the linear fit, indicating that RA is a robust model for predicting the compressive strength values of SCC.

The results of all performance measures, including R-value and MSE of the whole model with training, validation, and test, are finally summarized in Table 4. Therefore, these findings suggest that the Levenberg–Marquardt method is suitable for estimating the compressive strength of self-compacting concrete using recycled aggregates.

### 5.2. Bayesian Regularization

**Figure 12:**
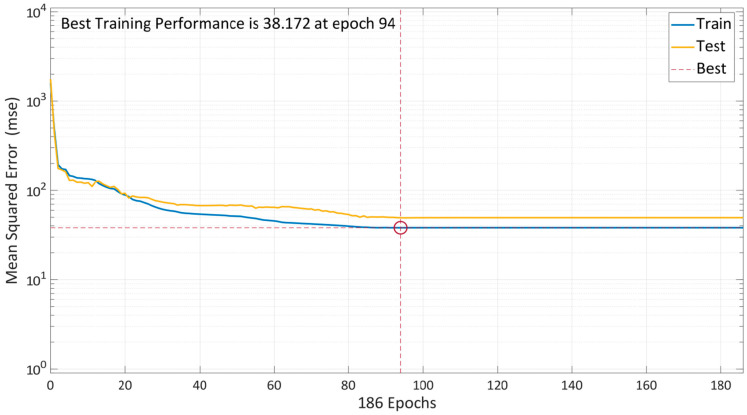
Model performance using BR algorithm.

Similarly, the Bayesian regularization approach is used to train the model. The model’s performance with the same number of neurons is depicted in Figure 12. The plot comprises of two-colored lines that only indicate training and testing because this algorithm already has an in-built kind of validation during the training stage. To avoid overfitting the data, the model starts with a high MSE and gradually lowers its reliance on the training parameters. The graph demonstrates that the model required several epochs since BR takes slightly longer time. After 94 epochs, the training and testing error lines had significantly decreased, and they were almost straight. The model is further trained to ensure comprehensive validation, and training is halted after 190 epochs. The model evaluated that the best training performance by the BR algorithm is at the 94th iteration, i.e., a minimum MSE of 38.172.

**Figure 13:**
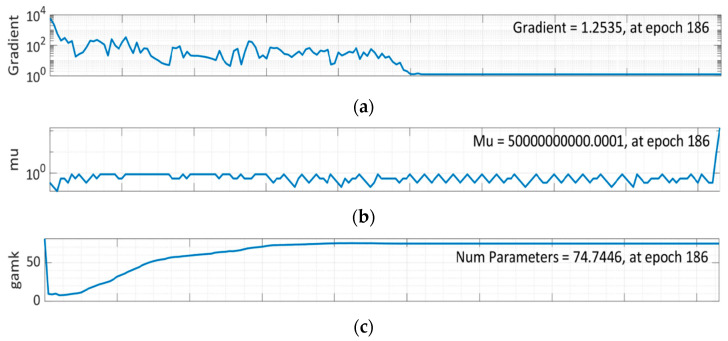

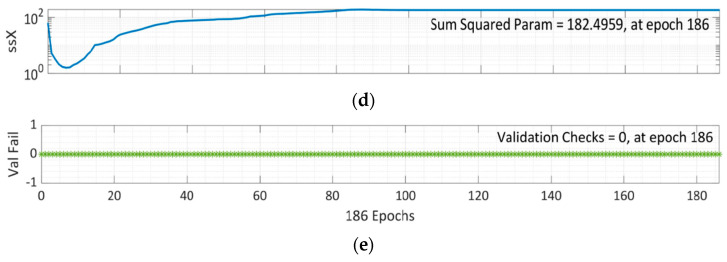
Training State of BR Algorithm: (**a**) Gradient graph at epoch 186; (**b**) Mu graph at epoch 186; (**c**) Number parameter graph at epoch 186; (**d**) S.S.X. graph at epoch 186; (**e**) Validation checks graph at epoch 186.

It can be seen from Figure 13a that training data errors decrease over time. Still, validation and test data errors rise. Therefore, the model is trained further at 186 epochs for comprehensive validation, and epoch 94 is found out to be the most suitable option for this network training, as shown in Figure 12. As Mu is the controlling parameter to train in the BR algorithm, 5 × 10^10^ was chosen after several rounds, as seen in Figure 13b. Effective parameters used by this algorithm were approximately 74 at epoch 186. Figure 13e further shows that no validation checks are carried out, since BR already has an in-built type of validation during the training stage, negating the need for a validation step.

**Figure 14:**
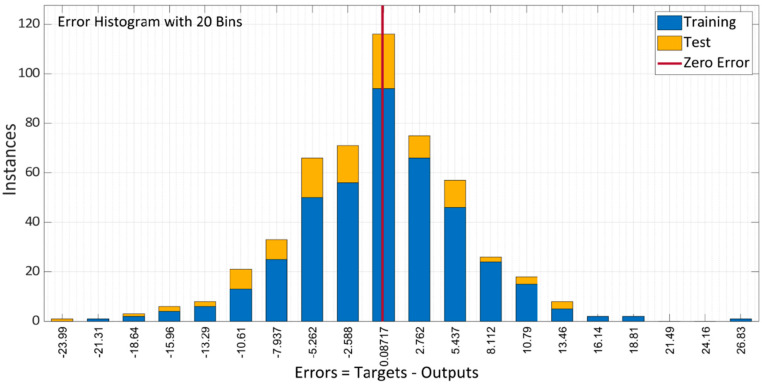
Error histogram of BR algorithm model.

Figure 14 depicts the model error histogram between training and testing. The plot illustrates that in comparison to the LM approach, the bar’s approach to the zero-error line is rather exceptional and the error is very low. The model does a decent job of forecasting the results of the compressive characteristics of SCC with RA, according to the results of this performance criteria.

**Figure 15:**
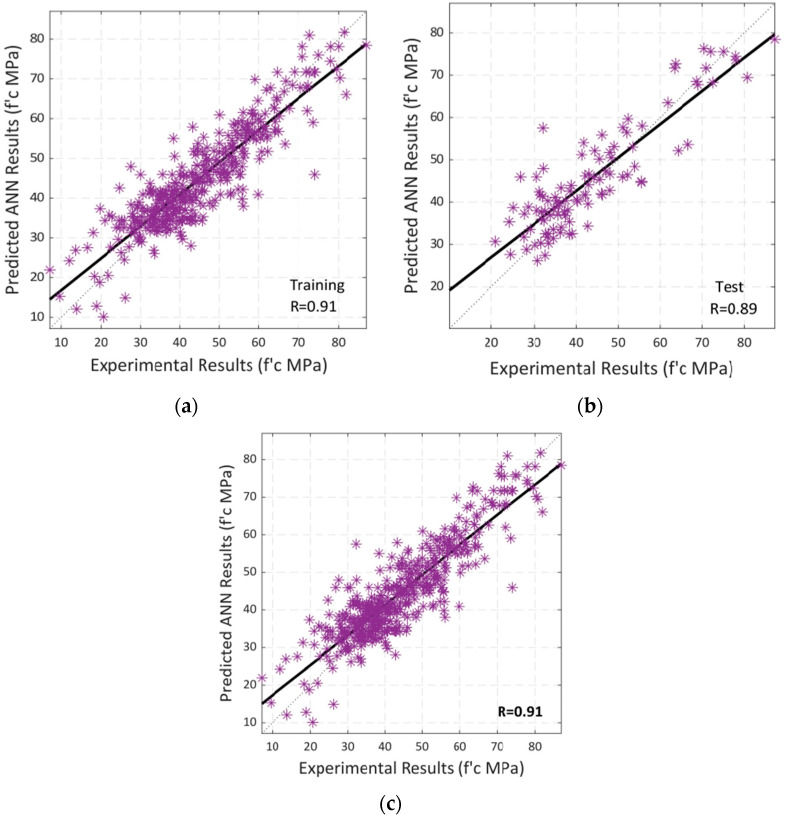
Regression analysis by using BR algorithm between experimental and predicted compressive strength: (**a**) Training; (**b**) Testing; (**c**) Overall Dataset.

Following that, a regression analysis was carried out in the same way. The training and testing correlations between the model’s input and output variables are shown in Figure 15a–c, which depict the overall correlation. A black-colored linear fit is presented in each scenario. The total R-value of 0.91 indicates that the model trained using Bayesian regularization has a high level of accuracy in predicting the output, i.e., the compressive strength of SCC using RA.

Table 5 concludes by summarizing all the results for the performance parameters, including the R-value and MSE of the entire model with training and testing. In general, our findings imply that Bayesian regularization may be used to calculate the compressive strength of self-compacting concrete constructed from recycled resources.

### 5.3. Scaled Conjugate Gradient Backpropagation

**Figure 16:**
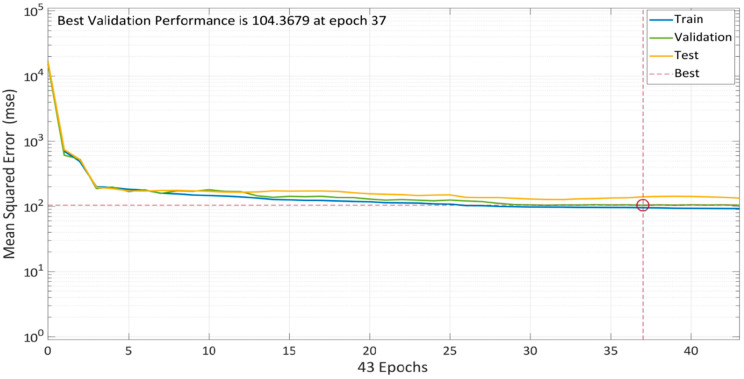
Model performance using SCG algorithm.

Scaled Conjugate Gradient Backpropagation (SCGB) is used for model training. The model’s performance with 10 total neurons is shown in Figure 16. Multiple colored lines make up the plot, which stand for training, validation, and testing. To avoid overfitting the data, the model starts with a high MSE and gradually lowers it dependent on the validation parameters. According to the graph, MSE did not significantly decrease when compared to the other two approaches. The training error decreased after 37 epochs; however, the validation and testing errors were somewhat rising. The model training finished after six more repetitions, and the optimized model with the lowest MSE was produced.

**Figure 17:**
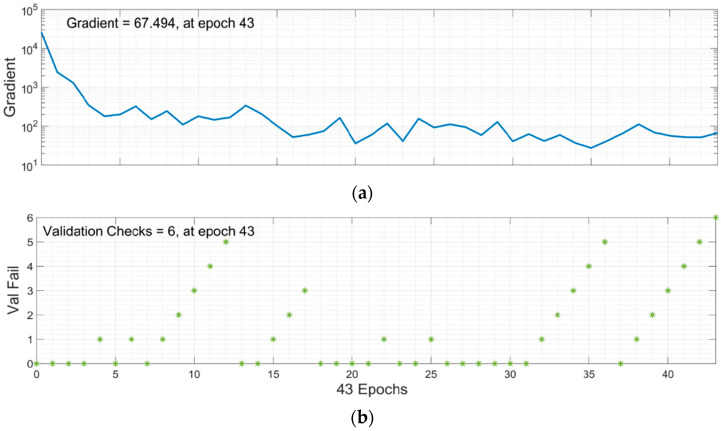
Training State of SCG Algorithm: (**a**) Gradient graph at epoch 43; (**b**) Validation checks graph at epoch 43.

Epoch 37 is the suitable option for this network training because while training data errors decrease over time, validation errors and test data increase. Figure 17b makes it clear that the training process was stopped after six validation failures.

**Figure 18:**
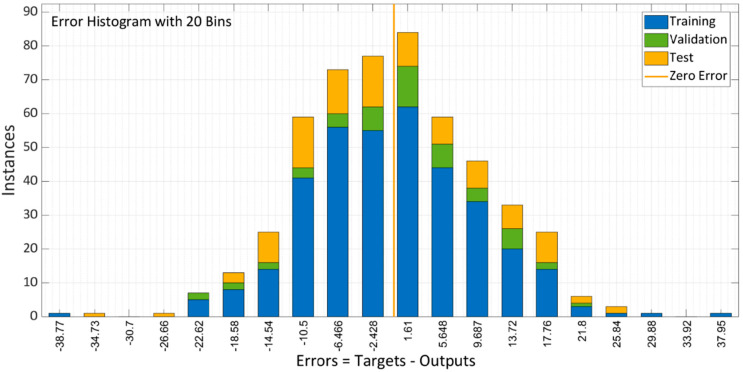
Error histogram of SCG algorithm model.

For training, validation, and testing, Figure 18 shows the model error histogram. The graph demonstrates how inaccurately the error bar bins seem to converge to the zero-error line. These results conclude that the model has high error values in comparison to LM and BR algorithms and performs badly in forecasting the results of SCC compressive strengths with RA.

**Figure 19:**
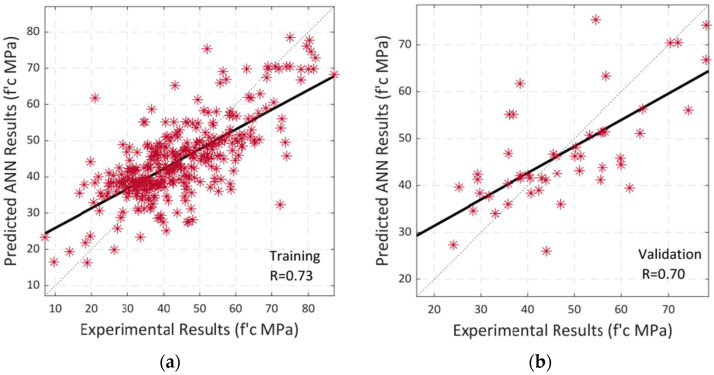

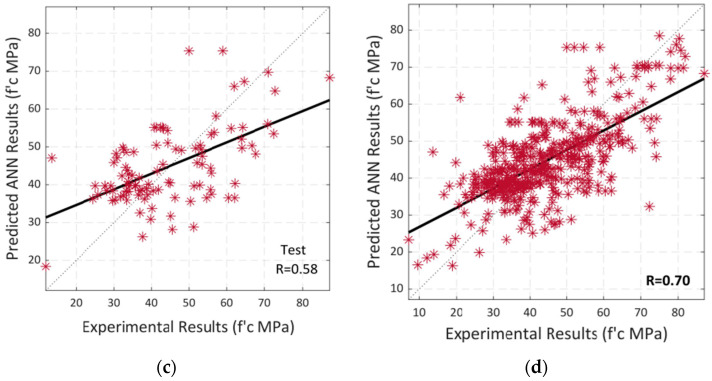
Regression analysis by using SCG algorithm between experimental and predicted compressive strength: (**a**) Training; (**b**) Validation; (c) Testing; (**d**) Overall Dataset.

Following that, a study of regression is then performed. Figure 19a–c illustrate the relationship between training, validation, and testing for the model’s input and output values. Figure 19 displays the model’s overall accuracy or correlation (d). A linear fit is shown in black in each instance. The total R-value of 0.64, which indicates a mediocre or average model for predicting SCC compressive strength using RA, should make it clear that their connection is not linear.

Table 6 presents the findings for all performance measures, including R-value and MSE for the whole model including training, validation, and testing. According to our research, the SCGB algorithm is less accurate than LM and BR in predicting the compressive strength of self-compacting concrete incorporating recycled aggregates.

### 5.4. Comparison of Algorithms

**Figure 20:**
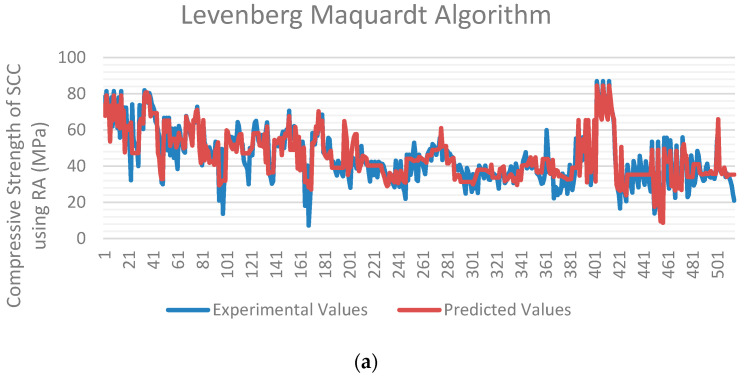

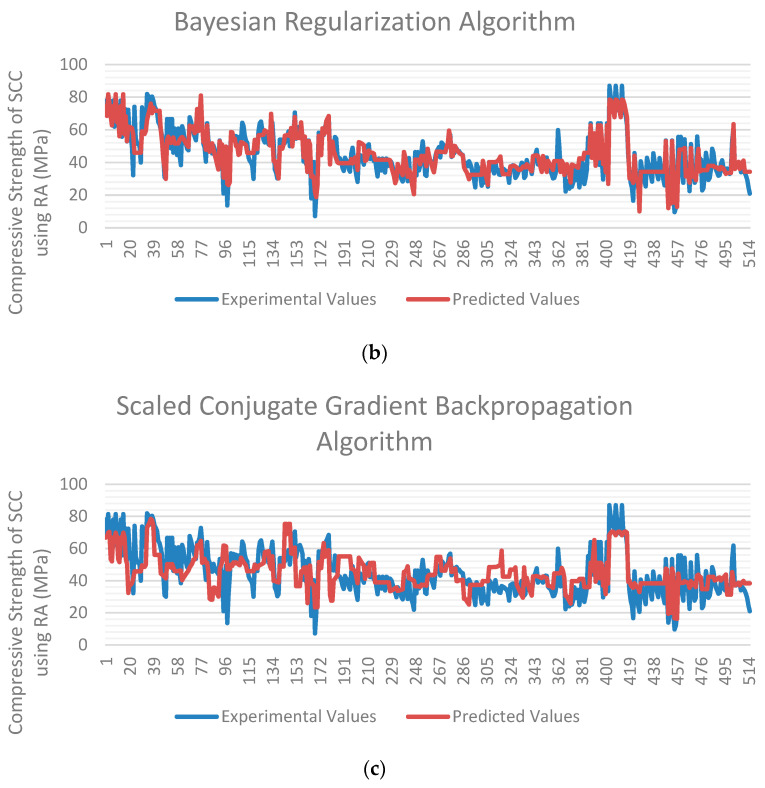
Comparison of compressive strength’s experimental and predicted values by different algorithms employed in ANN: (**a**) LM, (**b**) BR, and (**c**) SCG.

The three approaches were compared based on experimental data and ANN predictions. Figure 20a–c compare experimentally and predicted values of models trained using the LM, BR, and SCG algorithms, respectively. The red line on the *y*-axis represents projected values, whereas the blue line represents experimental values of SCC compressive strength using recycled aggregates. The data set of 515 samples is shown on the *x*-axis.

**Figure 21:**
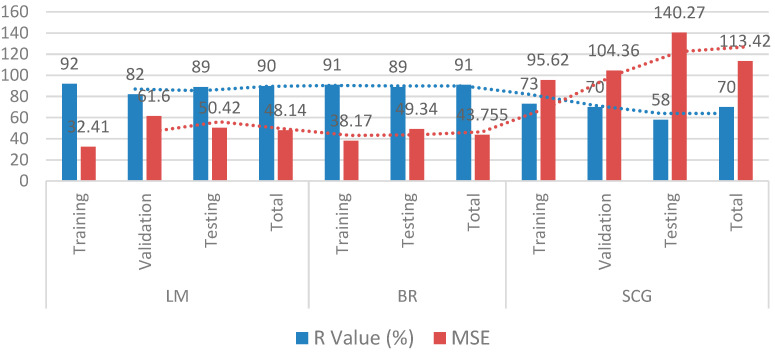
R-value (in % age) and MSE of LM, BR, and SCG algorithms.

The higher discrepancy between the two lines shows the greater error between the two parameters. The values predicted by LM and BR algorithms correlate well with the experimental values, as evident from the graphs. In contrast, a more significant difference between the two lines is indicated by the SCG algorithm. Figure 21 depicts the total R-value (in percentage) and mean squared error of all algorithms in graphical form.

As shown in Figure 20a–c and Figure 21, the Bayesian regularization and Levenberg–Marquardt algorithm both have nearly the same best fitting graphs, and both have roughly the same R-value and MSE. Given the variety of the data, the BR technique performed better because it can give significant generalization for complicated datasets [111]. It is also concluded that using the extensive current data set, the Levenberg–Marquardt algorithm has a high speed and nearly the same prediction rate as the BR algorithm and can predict the compressive strength of self-compacting concrete using recycled aggregates with high accuracy. The SCG algorithm showed poor results compared to the other two algorithms.

### 5.5. Sensitivity Analysis

Sensitivity analysis demonstrates how one single input variable influences the output variable. The impact of the input variables on the output variable increases with increasing sensitivity levels. The input variable has a sizable influence on the variables of output prediction, as claimed by Shang et al. [112]. Sensitivity analysis was conducted to assess the impact of one single input variable, including fine aggregate, cement, water, admixture, superplasticizer, and coarse aggregates, on the variability of compression strength of self-compacting concrete incorporating recycled aggregates. The sensitivity analysis is calculated using Equations (3) and (4), which are listed below.

(3) $$S_{i}=\frac{N_{i}}{\sum_{i=1}^{n}N_{i}}\times \;100$$

(4) $$N_{i}{=f}_{\max}\left(x_{i}\right)-f_{\min}\left(x_{i}\right),\;i=1,\ldots ,\;n$$

where f_max_(x_i_) and f_min_(x_i_) are the estimated maximum and minimum compressive strength in reference to the variables of input.

**Figure 22:**
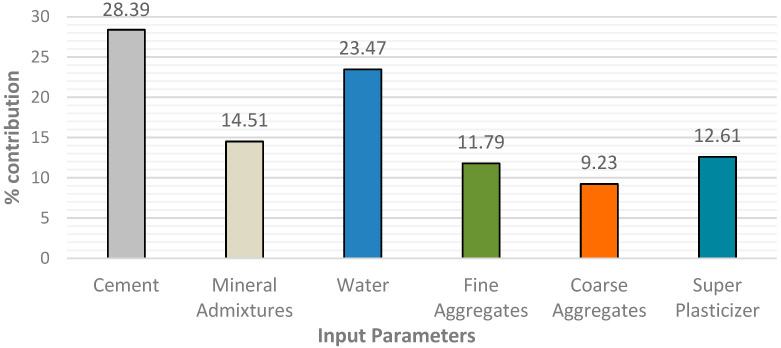
Contribution of input parameters to compressive strength of SCC with RA.

Fine aggregate, cement, water, admixture, superplasticizer, and coarse aggregate are all-important input factors in estimating the compressive strength of self-compacting concrete with recycled aggregate. Findings of this sensitivity study are shown in Figure 22, where it could be demonstrated that water and Portland cement are the critical input factors in determining the compressive strength of SCC with recycled aggregate. Portland cement makes up 28.39% of the total, while water makes up 24.37%. Shang et al. [112] said that Portland cement is a critical element in compressive strength prediction. However, the variable of input such as fine aggregates, admixture, and superplasticizer all show comparable contributions of 14.51%, 12.61%, and 11.79%, respectively. The study results revealed that CA (9.32%) is the least efficient variable in predicting compressive strength, which is consistent with prior research findings [113].

## 6. Conclusions

This research aims to predict and compare the compressive strength of self-compacting concrete (SCC) modified with recycled aggregates (RA) using three different artificial neural network (ANN) algorithms: LM, BR, and SCG. The six input parameters that train the model are cement, water, admixtures, coarse aggregates, fine aggregates, and superplasticizer. R-value and MSE were employed as measures for assessment. The following findings were obtained from this research.

1. In developing LM, BR, and SCG models, a total of 515 samples were acquired from research papers and randomly distributed into 70%, 10%, and 20% for training (360), validation (52), and testing (103), respectively. Due to the built-in validation mechanism in the training stage of the BR algorithm, the ratio became 80% for training and 20% for testing.
2. For the present study, three algorithms, LM, BR, and SCG, were trained and evaluated, giving an overall accuracy of 90%, 91%, and 70%, respectively, with MSE values of 48.14, 43.75, 113.42. The SCG algorithm is the worst model for forecasting SCC compressive strength, with RA having poor correlation and mean squared error.
3. Bayesian regularization gives better results than LM and SCG, with the highest coefficient of correlation (R = 91%) and the lowest MSE (43.75). However, in the meantime, the LM algorithm also gave nearly the same coefficient of correlation (R = 90%) with a much shorter processing time than the BR algorithm.
4. The findings demonstrated that the LM and BR algorithms are suitable models and can be adapted to predict the 28 days compressive strength of self-compacting concrete amended with recycled aggregates.
5. According to the model’s sensitivity analysis, the most significant parameter determining compressive strength is cement, contributing 28.39%. Water, with a contribution of 23.47%, is another crucial variable in predicting compressive strength in the same setting. The variable with the lowest occurrence, on the other hand, was coarse aggregate (9.23%). All the data suggest that cement and water improve the compressive strength of SCC with RA, but coarse aggregate reduces it. Admixture, fine aggregates, and superplasticizers, on the other hand, play a minor role in the development of the model.

## Figures and Tables

**Table 1:** Experimental data.

No	Reference	# Mix	% Data	No	Reference	# Mix	% Data
1	Ali et al., 2012 [49]	18	3.50	28	Nili et al. [50]	10	1.94
2	Aslani et al., 2018 [51]	15	2.91	29	Pan et al., 2019 [52]	6	1.17
3	Babalola et al., 2020 [53]	14	2.72	30	Pereira-de-Oliveira et al., 2014 [54]	4	0.78
4	Bahrami et al., 2020 [55]	10	1.94	31	Poongodi et al., 2020 [56]	9	1.75
5	Barroqueiro et al. [57]	6	1.17	32	Revathi et al., 2013 [58]	5	0.97
6	Behera et al., 2019 [59]	6	1.17	33	Revilla Cuesta et al., 2020 [60]	5	0.97
7	Bidabadi et al., 2020 [61]	11	2.14	34	Sadeghi-Nik et al., 2019 [62]	12	2.33
8	Chakkamalayath et al., 2020 [63]	6	1.57	35	Salesa et al., 2017 [64]	4	0.78
9	Duan et al., 2020 [65]	10	1.94	36	Sasanipour et al., 2019 [66]	10	1.94
10	Fiol et al., 2018 [67]	12	2.33	37	Señas et al., 2016 [9]	6	1.17
11	Gesoglu et al., 2015 [68]	24	4.66	38	Sharifi et al., 2013 [69]	6	1.17
12	Grdic et al., 2010 [70]	3	0.58	39	Ali and Al-Tersawy, 2014 [49]	15	2.91
13	Guneyisi et al., 2014 [71]	5	0.97	40	Silva et al., 2016 [72]	15	2.91
14	Guo et al., 2020 [73]	27	5.24	41	Singh et al., 2019 [74]	12	2.33
15	Kapoor et al., 2016 [75]	8	1.55	42	Sua-iam et al., 2013 [76]	20	3.88
16	Katar et al., 2021 [77]	4	0.78	43	Sun et al., 2020 [78]	10	1.94
17	Khodair et al., 2017 [79]	20	3.88	44	Surendar et al., 2021 [80]	7	1.36
18	Kou et al., 2009 [81]	13	2.52	45	Tang et al., 2016 [82]	5	0.97
19	Krishna et al., 2018 [83]	5	0.97	46	Thomas et al., 2016 [84]	4	0.78
20	Kumar et al., 2017 [85]	4	0.78	47	Tuyan et al., 2014 [86]	12	2.33
21	Li et al., [87]	4	0.78	48	Uygunoglu et al., 2014 [88]	8	1.55
22	Long et al., 2016 [89]	4	0.78	49	Wang et al., 2020 [90]	5	0.97
23	Mahakavi and Chithra, 2019 [91]	25	4.85	50	Yu et al., 2014 [92]	3	0.58
24	Manzi et al., 2017 [93]	4	0.78	51	Yu et al., 2020 [94]	3	1.14
25	Martínez-García et al., 2020 [95]	4	0.78	52	Yu et al., 2021 [96]	21	4.08
26	Mo et al., 2021 [97]	5	0.97	53	Zhou et al., 2013 [98]	6	1.17
27	Nieto et al., 2018 [99]	22	4.27		Total	515	100

**Table 2:** Minimum, Mean, and Maximum values of the input and output variables.

	Variables	Abbreviation	Minimum	Mean	Maximum
Input	Cement (kg/m^3^)	C	78.00	375.84	635.00
	Mineral Admixture (kg/m^3^)	MA	0.00	135.17	515.00
	Water (kg/m^3^)	W	45.50	176.87	277.00
	Fine Aggregates (kg/m^3^)	FA	532.20	845.10	1200.00
	Coarse Aggregates (kg/m^3^)	CA	328.00	784.91	1170.00
	Superplasticizer (kg/m^3^)	SP	0.00	4.50	16.00
Output	Compressive Strength (MPa)	f’c	7.17	44.94	87.00

**Table 3:** Data splitting for model’s training, validation, and testing.

Levenberg–Marquardt Algorithm		
Phase	Percentage (%)	No. of Specimens
Training	70	360
Validation	10	52
Testing	20	103
Total	100	515
**Bayesian Regularization**		
Training	80	412
Validation	-	0
Testing	20	103
Total	100	515
**Scaled Conjugate Gradient Backpropagation**		
Training	70	360
Validation	10	52
Testing	20	10
Total	100	515

**Table 4:** Summary of LM algorithm model assessment parameters.

Phase	Function	MSE	R
Training	trainLM	32.41	0.921
Validation	trainLM	61.60	0.823
Testing	trainLM	50.42	0.890
Total	trainLM	48.14	0.902

**Table 5:** Summary of BR algorithm model assessment parameters.

Phase	Function	MSE	R
Training	trainbr	38.17	0.9154
Testing	trainbr	49.34	0.8923
Total	trainbr	43.755	0.9100

**Table 6:** Summary of SCGB algorithm model assessment parameters.

Phase	Function	MSE	R
Training	trainSCG	95.62	0.732
Validation	trainSCG	104.36	0.704
Testing	trainSCG	140.27	0.581
Total	trainSCG	113.42	0.701

## Data Availability

The data are available by request from the corresponding author.
